# Single-cell transcriptomics identifies ergothioneine as a mitochondrial protector to prevent AKI-to-CKD progression

**DOI:** 10.1371/journal.pone.0351630

**Published:** 2026-06-23

**Authors:** Jiaxin Peng, Jing Chen, Zhipu Qian, Mingzhang Han, Ling Chen

**Affiliations:** Department of Internal Medicine and Geriatrics, Zhongnan Hospital of Wuhan University, Wuhan University, Wuhan, Hubei, China; Universita degli Studi della Campania Luigi Vanvitelli, ITALY

## Abstract

This study investigates the role and mechanism of ergothioneine (EGT) in mitigating the progression of acute kidney injury (AKI) to chronic kidney disease (CKD). Using a cisplatin-induced mouse model of the AKI-to-CKD transition with EGT intervention, we combined histopathological examination, biochemical assays, and single-cell RNA sequencing (scRNA-seq) to provide evidence that EGT may improve renal function parameters and attenuate renal injury and fibrosis. scRNA-seq analysis revealed that EGT was associated with partial normalization of mitochondria-related gene expression in renal tubular epithelial cells, accompanied by enrichment of oxidative phosphorylation and electron transport chain pathways. Furthermore, our in vitro experiments supported a protective association of EGT with mitochondrial injury-related phenotypes in injured renal tubular epithelial cells, as indicated by reduced reactive oxygen species generation, partial preservation of mitochondrial membrane potential, and increased cellular ATP levels. These findings suggest that EGT may attenuate AKI-to-CKD progression in association with improved mitochondrial homeostasis, offering a potential therapeutic strategy for kidney diseases.

## 1. Introduction

Acute kidney injury (AKI) is a common clinical syndrome characterized by a rapid decline in renal function, often resulting from ischemic, toxic, or septic insults [1]. Despite initial recovery, a significant proportion of AKI patients progress to chronic kidney disease (CKD), a condition marked by persistent renal dysfunction, fibrosis, and irreversible structural damage [2]. This maladaptive AKI-to-CKD transition has emerged as a critical pathway contributing to the growing population of patients with end-stage renal disease [3]. It represents a major global health burden due to its high incidence, particularly in hospitalized patients, and its association with substantial morbidity and mortality. However, the underlying molecular and cellular mechanisms remain incompletely elucidated. Importantly, effective therapeutic strategies to halt or slow this progression are strikingly limited, highlighting an urgent need for deeper mechanistic insight and novel interventions [4].

Mitochondrial dysfunction has been increasingly recognized as a pivotal mechanism in the pathogenesis of both AKI and CKD [5]. Renal tissue cells are abundantly enriched with mitochondria, second only to cardiomyocytes, which is essential for generating substantial ATP via oxidative phosphorylation to meet the high energy demands required for renal cellular functions [6]. In AKI, insults such as ischemia or toxins induce mitochondrial damage, leading to impaired ATP synthesis, excessive reactive oxygen species (ROS) production, and activation of apoptotic pathways [7]. These mitochondrial abnormalities not only contribute to acute cell death but also promote maladaptive repair, persistent inflammation, and fibrotic responses, thereby facilitating the transition to CKD [8]. Some studies have revealed that in the AKI microenvironment, persistent mitochondrial damage leads to inadequate energy supply in renal tubular epithelial cells, resulting in a metabolic shift from fatty acid β-oxidation (FAO) to glycolysis. This transition represents a double-edged sword [9]. On one hand, enhanced glycolysis acts as a compensatory mechanism for ATP generation; on the other hand, chronic suppression of FAO coupled with sustained glycolytic activation contributes to impaired repair of renal tubular epithelial cells, and promotes inflammation, lipid accumulation, and fibrosis, thereby facilitating the progression from AKI to CKD. Restoration of mitochondrial integrity and bioenergetics has thus emerged as a promising therapeutic strategy to interrupt this progression.

Ergothioneine (EGT) is a natural amino acid and thiol antioxidant widely distributed in different organisms [10]. It exhibits potent antioxidant and anti-inflammatory properties. It has been shown to protect against various models of organ injury [11,12]. Previous studies have demonstrated that EGT binds to and activates MPST, enhancing mitochondrial respiration and energy metabolism, which consequently improves endurance exercise performance in mice [13]. Furthermore, EGT has been shown to alleviate mitochondrial impairment through regulation of the AMPK/SIRT1/PGC-1α signaling pathway, thereby reducing neuroinflammation in aging mouse models [14]. In a rat model of diabetic nephropathy, EGT induces Nrf2 activation and upregulates antioxidant cytoprotective genes (HO-1 and NQO1), thereby enhancing the antioxidant defense system and ameliorating renal dysfunction [15]. However, its role in mitigating the progression from AKI to CKD, particularly through the preservation of mitochondrial function in specific renal cell types, remains largely unexplored.

In this study, we employed a cisplatin-induced model of AKI-to-CKD progression to evaluate the therapeutic potential of EGT. Utilizing a multidisciplinary approach combining histopathological examination, biochemical assays, and single-cell RNA sequencing (scRNA-seq), we sought to determine whether EGT intervention ameliorates renal functional impairment, attenuates structural damage, and reduces fibrosis. Furthermore, since renal tubular epithelial cells are the central cell type affected by AKI [16], we aimed to investigate whether EGT was associated with improved mitochondria-related transcriptional and injury-related phenotypic features in key renal tubular segments, including distal convoluted tubules (DCT), loops of Henle (LOH), and proximal tubule (PT) cells. An overview of the experimental design is provided as a graphical abstract in the S1 Fig. Through these analyses, we aimed to clarify the potential contribution of EGT-associated mitochondrial protection to AKI-to-CKD progression and to assess its translational relevance as a therapeutic candidate.

## 2. Materials and methods

### 2.1. Chemicals and reagents

Cisplatin (B24462, Purity≥98%) was obtained from Shanghai Yuanye Biotechnology Co., Ltd. (Shanghai, China) and dissolved in PBS. L-(+)-Ergothioneine (EGT, L134175, Purity≥98%) was purchased from Shanghai Aladdin Biochemical Technology Co., LTD (Shanghai, China). Recombinant human TGF-β1 (240-b-002) was purchased from R&D Systems (Minneapolis, MN, USA).

### 2.2. Animals and experiment design

Six-week-old male C57BL/6 mice were used for all experiments and randomly divided into three groups after a one-week adaptation period. (1) Control group (n = 6): mice received intraperitoneal injection of saline. (2) AKI-CKD group (n = 6): the cisplatin-induced AKI-to-CKD transition model was established by weekly intraperitoneal cisplatin injections (8 mg/kg) for four weeks, following the previously described method [17]. (3) AKI-CKD + EGT group (n = 6): mice received weekly intraperitoneal cisplatin injections with continuous EGT supplementation (0.5 mg/mL in drinking water, ~ 100 mg/kg/day) initiated 3 days before cisplatin administration until study completion. The dose and duration of EGT administration were chosen based on previous studies. After 8 weeks, mice were euthanized by cervical dislocation under deep anesthesia following ethical guidelines. Anesthesia was induced by intraperitoneal injection of sodium pentobarbital (50 mg/kg) prior to euthanasia to minimize suffering. Throughout the study, all animals were monitored daily for signs of distress, including weight loss, piloerection, and reduced mobility. Humane endpoints were applied, and mice showing signs of severe illness, such as a > 20% weight loss or an inability to reach food or water, were promptly euthanized using the same method. Blood and urine were collected pre-euthanasia. Kidneys were weighed and divided longitudinally into two halves; one half was preserved in 4% paraformaldehyde and paraffin-embedded for histological analysis and the other half was used for single-cell RNA sequencing. This study was approved by the Animal Ethics Committee of Zhongnan Hospital of Wuhan University (approval number: WP20220486).

### 2.3. Body weight and kidney weight

Throughout the study period, body weights of all mice were recorded weekly with an electronic precision scale (FA31002C, Sartorius, Germany). At the study endpoint, kidneys were harvested from each mouse and weighed for subsequent analysis.

### 2.4. Plasma and urine analysis

Renal function was assessed by measuring serum creatinine (Cre) and blood urea nitrogen (BUN) levels using an automatic biochemical analyzer (Hitachi Instruments Service Co., Ltd., Tokyo, Japan). Additionally, urinary microalbumin and creatinine concentrations were determined using the same automated analyzer system.

### 2.5. Hematoxylin & Eosin staining

For histopathological evaluation, kidney tissues were fixed in 4% paraformaldehyde, embedded in paraffin, and sectioned. Sections were dewaxed in xylene and rehydrated through graded alcohols. The staining procedure involved hematoxylin treatment for 2 minutes followed by a 3-minute tap water rinse, brief differentiation in hydrochloric acid-alcohol for 10 seconds, eosin counterstaining for 10 seconds, and a final 10-minute wash under running tap water. Stained sections were dehydrated, cleared, and mounted for microscopic examination (Olympus, Tokyo, Japan).

### 2.6. Masson’s trichrome staining

For fibrosis assessment, kidney tissue sections were subjected to Masson’s trichrome staining. The samples were stained with Weigert’s Iron Hematoxylin for 10 minutes, followed by tap water rinsing and brief differentiation in 1% acid alcohol (10–20 seconds) with subsequent 1-minute running water wash. Sections were then stained with scarlet-acid fuchsin for 5 minutes and rinsed in distilled water. Finally, sections were differentiated in phosphomolybdic acid solution followed by 5-minute incubation in aniline blue solution. Stained sections were examined and imaged using an Olympus microscope (Olympus, Tokyo, Japan).

### 2.7. Single-cell RNA-seq raw data processing

The study analyzed single-cell RNA sequencing data encompassing 216,680 cells from three experimental groups (Control, AKI-CKD, and AKI-CKD + EGT), with three biological replicates per group. The UMI count matrix was converted into a Seurat object by the R package Seurat (version 4.3.0) [18]. Cells with UMI numbers <1000 or with detected genes < 500 or with over 15% mitochondrial-derived UMI counts were considered low-quality cells and were removed, ultimately retaining 169,064 cells. Additional filtering removed genes expressed in fewer than five cells. Following quality control, the count matrices from multiple samples were integrated using Seurat to enable comparative analysis across samples. Particularly, the merged count matrices were log-normalized by ‘NormalizeData’ function and scaled by ‘ScaleData’ function. Subsequently, the top 2000 most variable genes were selected through the ‘FindVariableFeatures’ function, and principal components (PCs) were derived via ‘RunPCA’ function. Data integration was then performed using the harmony algorithm [19]. Nearest neighbors were computed based on the top 30 PCs, followed by cluster identification employing the ‘FindClusters’ function with a resolution parameter of 1.1. Clustering results were visualized using tSNE or UMAP plot. For cell type annotation, we first identified cluster-specific marker genes using Seurat’s ‘FindMarkers’ function, then mapped these markers to known cell types using the ScType reference database.

### 2.8. Differential gene expression analysis

Differentially expressed genes (DEGs) were identified using the FindMarkers/ FindAllMarkers function from the Seurat package (one-tailed Wilcoxon rank sum test, P values adjusted for multiple testing using the Bonferroni correction). DEGs were identified using the following thresholds: absolute log fold change ≥ 0.5, difference in the percentage of expressing cells ≥ 0.2, and adjusted P value < 0.05.

### 2.9. Transcription factor regulatory network analysis

Transcription factor (TF) modules were identified using the SCENIC Python workflow (version 0.11.2) using default parameters (http://scenic.aertslab.org)[20]. A mouse TF gene list was used from the resources of pySCENIC (https://github.com/aertslab/pySCENIC/tree/master/resources). Activated TFs were identified in the AUC matrix, and differentially activated TFs were selected using R package limma [21]. To identify cluster-specific regulons (especially for analyses with many cell types, where some regulons are common to multiple of them) we used the Regulon Specificity Score (RSS) [22]. Networks of the modules with TFs and their target genes were visualized by Cytoscape (v3.9.1) (https://cytoscape.org/).

### 2.10. Functional enrichment analysis

Functional annotation of genes was performed through Gene Ontology (GO) and KEGG pathway analysis using KOBAS 2.0 [23]. Statistical significance of term enrichment was determined by hypergeometric testing with Benjamini-Hochberg false discovery rate (FDR) correction.

### 2.11. Cell culture

The human renal proximal tubular epithelial cell line HK-2 was obtained from the China Center for Type Culture Collection (CCTCC, Wuhan, China). This cell line was cultured in MEM medium supplemented with 10% fetal bovine serum and 1% penicillin-streptomycin and maintained in a cell culture incubator (Binder, Tuttlingen, Germany) at 37°C with 5% CO_2_.

### 2.12. Transmission electron microscopy

After obtaining kidney tissues, they were immediately fixed with electron microscopy fixative (Servicebio, Wuhan, China). Following a series of processing steps, morphological changes in mitochondria were observed using a transmission electron microscope (HT7800, Hitachi, Japan).

### 2.13. Detection of reactive oxygen species (ROS) levels

HK-2 cells in the logarithmic growth phase were seeded into 6-well plates and divided into the following groups: control group, TGF-β (2 ng/ml) group, and TGF-β (2 ng/ml) + EGT (400 μmol/L) group. TGF-β-treated HK-2 cells were used as an in vitro model of maladaptive tubular stress and profibrotic signaling to evaluate whether EGT could alleviate mitochondria-related injury responses in renal tubular epithelial cells. After 24 hours, the cells were treated with culture medium containing the aforementioned drugs for 48 hours. Subsequently, the cells were incubated with the DCFH-DA (S0033S, Beyotime, China) probe working solution for 30 minutes. Subsequently, the cells were washed three times with serum-free medium, and the intracellular fluorescence intensity of each group was observed using an inverted fluorescence microscope.

### 2.14. Mitochondrial membrane potential

HK-2 cells in the logarithmic growth phase were seeded into 6-well plates, divided into three groups and subjected to the corresponding treatments. Following the protocol of the mitochondrial membrane potential assay kit with JC-1 (C2003S, Beyotime, China), cells were stained under light-protected conditions. Finally, green JC-1 monomers and red JC-1 aggregates were observed using an inverted fluorescence microscope.

### 2.15. ATP level measurement

HK-2 cells in the logarithmic growth phase were seeded into 6-well plates, divided into three groups and subjected to the corresponding treatments. After washing with PBS, cells in each group were lysed by adding 200 µL of lysis buffer per well, followed by thorough homogenization and complete lysis using an ultrasonic disruptor. The bioluminescence intensity was measured with a microplate reader equipped with a luminescence detection function. Meanwhile, protein concentrations in each group were quantified using the BCA assay. The ATP content in the test samples was then calculated accordingly.

### 2.16. Quantitative real-time PCR

Total RNA was extracted from HK-2 cells using TRIzol reagent according to the manufacturer’s instructions. RNA concentration and purity were assessed using a spectrophotometer. Complementary DNA was synthesized using a reverse transcription kit. Quantitative real-time PCR was performed using SYBR Green PCR Master Mix on a real-time PCR detection system. The relative mRNA expression levels of target genes were calculated using the 2^−ΔΔCt^ method and normalized to GAPDH. The primer sequences used in this study were as follows: UQCR11 forward, 5′-GCTACCGGGAGCTGGTCAAG-3′; UQCR11 reverse, 5′-GTCCAGGATCAGCCGCCAAT-3′; COX6B1 forward, 5′-CTACAAGACCGCCCCTTTTGA-3′; COX6B1 reverse, 5′-GCAGAGGGACTGGTACACAC-3′; GAPDH forward, 5′-GAGAAGGCTGGGGCTCATTT-3′; and GAPDH reverse, 5′-AGTGATGGCATGGACTGTGG-3′.

### 2.17. Statistical analysis

All data are presented as means ± standard deviation (SD). Statistical analyses were performed using GraphPad Prism 8.0.1 software (San Diego, CA, USA). Student’s t-test was used for comparisons between two groups. One-way analysis of variance (ANOVA) with Tukey’s correction was used for comparisons between multiple groups. The pheatmap package (https://cran.r-project.org/web/packages/pheatmap/index.html) in R was used to perform the clustering based on Euclidean distance. *P* < 0.05 was considered statistically significant. The graphical abstract was created with BioGDP [24] and is provided in the S1 Fig.

## 3. Results

### 3.1. EGT treatment delayed AKI-to-CKD progression

**Fig 1:**
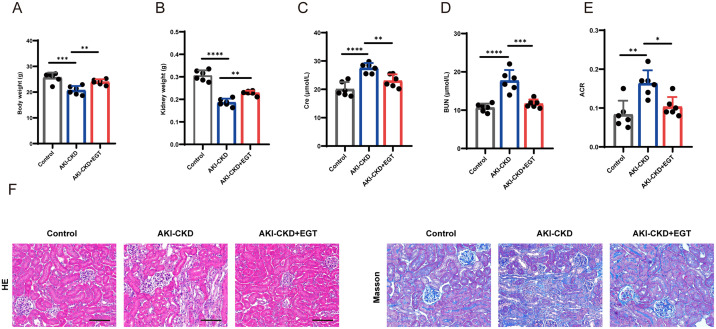
Effect of EGT on renal function and renal histopathologic injury in mice. **(A)** Body weight. **(B)** Kidney weight. **(C)** Cre. **(D)** BUN. **(E)** ACR. **(F)** Representative HE and Masson staining images. Scale bar = 100 μm. Data are expressed as mean ± SD, n = 6 mice per group, *P < 0.05; **P < 0.01; ***P < 0.001; ****P < 0.0001.

After 8 weeks of modeling, both body weight (Fig 1A) and kidney weight (Fig 1B) were significantly decreased in the AKI-CKD group compared with the control group. Serum Cre (Fig 1C), BUN (Fig 1D), and urinary albumin-to-creatinine ratio (ACR; Fig 1E) were significantly increased in AKI-CKD mice compared with controls, indicating impaired renal function. Notably, these pathological changes were attenuated by EGT treatment (Fig 1A-E). Compared with the control group, HE and Masson staining revealed kidney injury, inflammatory infiltration, and subsequent interstitial fibrosis in kidney tissues from AKI-to-CKD mice. Notably, these histopathological changes were attenuated by EGT treatment (Fig 1F).

### 3.2. Ergothioneine targets renal cell heterogeneity to mitigate AKI-to-CKD progression

**Fig 2:**
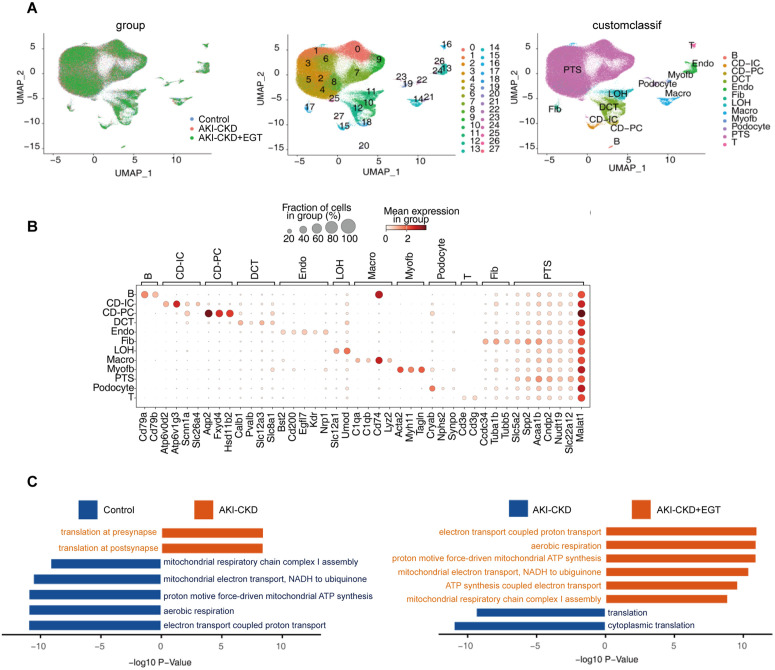
Cellular diversity in mouse kidneys delineated by single-cell transcriptomic analysis. **(A)** UMAP plot of single-cell transcriptomic profiles from Control, AKI-CKD, and AKI-CKD + EGT samples. Colors indicate cell clusters and annotations. **(B)** Dot plot showing expression of representative genes in each cell type. B, B cells; CD-IC, connecting tubule-intercalated cell; CD-PC, connecting tubule-principal cell; DCT, distal convoluted tubule; Endo, endothelial cells; Fib, fibroblast; LOH, loop of Henle; Macro, macrophages; Myofb, myofibroblast; PTS, segment of the proximal tubule; Podocyte, podocyte; T, T cells. **(C)** Bar plots showing the most enriched top 10 GO biological process results of upregulated and downregulated genes in AKI-CKD vs Control comparison and AKI-CKD + EGT vs AKI-CKD comparison.

To investigate the pathological mechanisms underlying AKI-to-CKD progression and the therapeutic mechanisms of ergothioneine, we performed single-cell RNA sequencing (scRNA-seq) on renal tissues from three groups of mice: the control group (control, n = 3), the model group (AKI-CKD, n = 3), and the model plus ergothioneine treatment group (AKI-CKD + EGT, n = 3). After quality control, we obtained transcriptomic profiles for a total of 169,064 cells. The single-cell transcriptome expression matrix of these 169,064 cells was normalized, subjected to principal component analysis (PCA) for dimensionality reduction, and the top 50 principal components were selected for UMAP dimensionality reduction and visualization. Through unbiased clustering analysis, we identified 28 cell subpopulations. Based on the characteristic marker genes of these clusters and referencing established marker gene databases from published literature, these subpopulations were annotated as 12 distinct cell types (Fig 2A). The relative expression of marker genes used for cell type annotation across different cells is shown in Fig 2B. To investigate the transcriptomic changes among the three groups at the whole-organ level, we analyzed the functional enrichment of differentially expressed genes (DEGs) identified from the combined analysis of all cell types. Comparative analysis between the AKI-CKD and control groups revealed that upregulated DEGs were primarily enriched in presynaptic and postsynaptic translation processes, while downregulated DEGs showed significant enrichment in mitochondrial-related biological processes, including mitochondrial respiratory chain complex Ⅰ assembly, mitochondrial electron transport from NADH to ubiquinone, proton motive force-driven mitochondrial ATP synthesis, aerobic respiration, and electron transport coupled to proton transport. Similarly, in the AKI-CKD + EGT versus AKI-CKD comparison, upregulated DEGs were again enriched in both the aforementioned mitochondrial pathways and additional cytoplasmic translation-related processes, whereas downregulated DEGs demonstrated enrichment in synaptic translation processes. These findings collectively highlight distinct patterns of transcriptional regulation associated with disease progression and therapeutic intervention (Fig 2C).

### 3.3. EGT treatment is associated with partial preservation of mitochondria-related transcriptional programs in DCT cells during AKI-to-CKD progression

**Fig 3:**
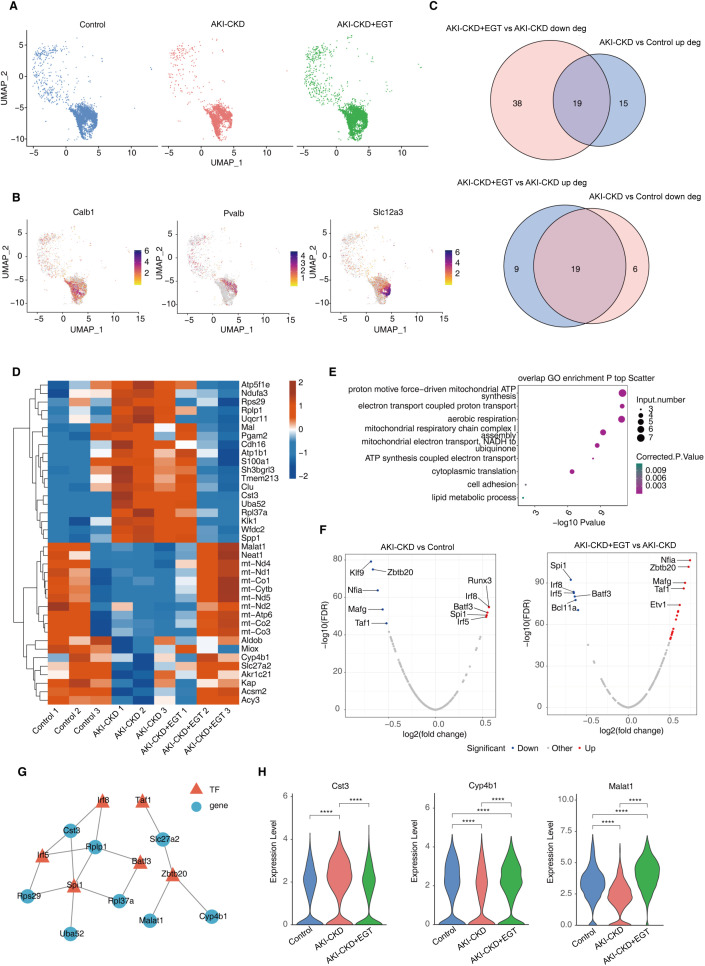
EGT treatment is associated with partial preservation of mitochondria-related transcriptional programs in DCT cells during AKI-to-CKD progression. **(A)** UMAP plot of DCT cells. Colors indicate sample groups. **(B)** UMAP plot showing the expression levels of marker genes in DCT cells. **(C)** The Venn diagram shows overlapping results of downregulated genes in AKI-CKD + EGT vs AKI-CKD and upregulated genes in AKI-CKD vs Control, upregulated genes in AKI-CKD + EGT vs AKI-CKD and downregulated genes in AKI-CKD vs Control. **(D)** The heatmap diagram showing the expression profile of overlapped DEGs in Fig 3C. **(E)** The bubble plot showing the most enriched GO results of overlapped DEGs in Fig 3C. **(F)** The volcano plot showing differential transcription regulons in two comparison groups (AKI-CKD vs Control and AKI-CKD + EGT vs AKI-CKD). Blue represents down-regulation, red represents up-regulation. **(G)** Network showing six DE-TFs and their target DEGs. **(H)** Violin plot showing the expression levels of Cst3, Cyp4b1, and Malat1 in each sample group in DCT cells.

To elucidate the mechanism by which EGT-targeted DCT cells delay AKI-to-CKD progression, we further analyzed isolated DCT cells. UMAP analysis revealed distinct spatial distribution differences in DCT cells between the AKI-CKD and Control groups, with partial restoration toward the normal state following EGT treatment (Fig 3A). The UMAP distribution of their marker genes, Calb1, Pvalb, and Slc12a3, is displayed in Fig 3B. Differential gene expression analysis of DCT cells across the three groups was performed. Volcano plots illustrating DEGs in AKI-CKD vs. Control and AKI-CKD + EGT vs. AKI-CKD are presented in S2A Fig. Functional enrichment analysis demonstrated that in AKI-CKD vs. Control, upregulated genes were associated with biological processes such as translation, mitochondrial electron transport chain, sodium ion homeostasis, and calcium ion homeostasis, whereas downregulated genes were enriched in respiratory electron transport, mitochondrial respiratory chain complex assembly, and aerobic respiration (S2B Fig). For AKI-CKD + EGT vs. AKI-CKD, upregulated genes were linked to mitochondrial electron transport, mitochondrial respiratory chain complex assembly, and fructose metabolism, while downregulated genes were involved in translation, aerobic respiration, and notably, also mitochondrial respiratory chain complex assembly (S2B Fig). This observation can be explained by the complexity of the mitochondrial respiratory chain assembly process itself, which involves dozens of genes encoding structural subunits, assembly factors, and regulatory proteins [25,26]. In the dysregulated state of AKI-CKD, the expression of these components becomes uncoordinated, with some genes upregulated and others downregulated. Consequently, when performing pathway enrichment analysis, both the upregulated subset and the downregulated subset of genes can independently map to the same broad biological pathway. EGT treatment was associated with recalibration of this imbalanced expression profile toward a more homeostatic transcriptional state. Notably, compared with the Control group, 19 genes were downregulated in the AKI-CKD group and subsequently upregulated after EGT treatment, whereas another 19 genes were upregulated in AKI-CKD and downregulated post-EGT (Fig 3C). These genes may represent key targets through which EGT mitigates AKI-to-CKD progression. A heatmap depicting their expression patterns across the three groups is shown in Fig 3D. Functional enrichment revealed that these genes participate in proton motive force-driven mitochondrial ATP synthesis, electron transport-coupled proton transfer, aerobic respiration, mitochondrial respiratory chain complex assembly, mitochondrial electron transport (NADH to ubiquinone), ATP synthesis coupled to electron transport, cytoplasmic translation, cell adhesion, and lipid metabolic processes (Fig 3E). These differential gene expression patterns suggest that AKI-to-CKD progression is associated with dysregulation of mitochondria-related transcriptional programs in DCT cells, whereas EGT treatment is associated with partial normalization of these transcriptional alterations. To investigate potential transcriptional regulators of these mitochondria-related genes, we analyzed differentially expressed transcription factors (TFs) in DCT cells. Notably, Zbtb20, Mafg, and Taf1 exhibited an initial upregulation followed by downregulation across the Control, AKI-CKD, and AKI-CKD + EGT groups, whereas Spi1, Irf5, Irf8, and Batf3 showed an opposite trend of initial downregulation followed by upregulation (Fig 3F). Further analysis of their target genes revealed a regulatory network (Fig 3G). Among these targets, Cst3, Cyp4b1, and Malat1 were closely associated with oxidative phosphorylation, and their specific expression trends across the three groups are shown in Fig 3H. Cst3 encodes cystatin C (CYSC), a well-established biomarker for estimating glomerular filtration rate (eGFR), the gold standard for renal function assessment [27]. Cyp4b1, a member of the cytochrome P450 enzyme family, is involved in fatty acid oxidation and related metabolic processes. Some cytochrome P450 enzymes have been implicated in mitochondrial dysfunction [28]. MALAT1 has been linked to mitochondrial structural and functional alterations, including mitochondrial copy number, oxidative phosphorylation, and mitophagy [29]. In summary, our findings suggest that DCT cells in AKI-to-CKD mice exhibit mitochondria-related transcriptional dysregulation, and that EGT treatment may partially preserve mitochondrial homeostasis in this tubular cell population.

### 3.4. EGT treatment is associated with partial preservation of mitochondria-related transcriptional programs in LOH cells during AKI-to-CKD progression

**Fig 4:**
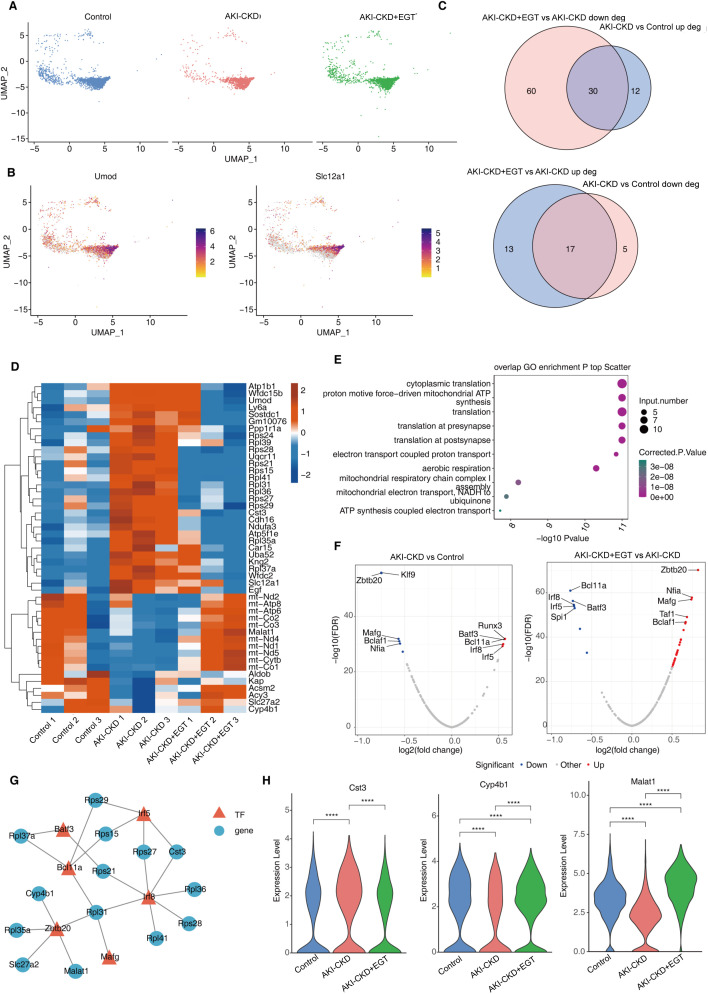
EGT treatment is associated with partial preservation of mitochondria-related transcriptional programs in LOH cells during AKI-to-CKD progression. **(A)** UMAP plot of LOH cells. Colors indicate sample groups. **(B)** UMAP plot showing the expression levels of marker genes in LOH cells. **(C)** The Venn diagram shows overlapping results of down-regulated genes in AKI-CKD + EGT vs AKI-CKD and up-regulated genes in AKI-CKD vs Control, up-regulated genes in AKI-CKD + EGT vs AKI-CKD and down-regulated genes in AKI-CKD vs Control. **(D)** The heatmap diagram showing the expression profile of overlapped DEGs in **C. (E)** The bubble plot showing the most enriched GO results of overlapped DEGs in **C. (F)** Volcano plot showing differential transcription regulons in two comparison groups (AKI-CKD vs Control and AKI-CKD + EGT vs AKI-CKD). Blue represents down-regulation, red represents up-regulation. **(G)** Network showing six DE-TFs and their target DEGs. **(H)** Violin plot showing the expression levels of Cst3, Cyp4b1, and Malat1 in each sample group in LOH cells.

To investigate the mechanism by which EGT affects LOH cells during AKI-to-CKD progression, we isolated LOH cells for further analysis. UMAP analysis revealed a significant reduction in LOH cell numbers in the AKI-CKD group, while EGT treatment partially restored the LOH cell population (Fig 4A). The UMAP distribution of LOH marker genes, Umod and Slc12a1, is shown in Fig 4B. Differential gene expression analysis of LOH cells was performed between AKI-CKD vs Control and AKI-CKD + EGT vs AKI-CKD groups, with volcano plots presented in S3A Fig. Functional enrichment analysis demonstrated that in AKI-CKD vs Control comparisons, upregulated genes were primarily associated with translation, mitochondrial electron transport chain, sodium ion homeostasis, and ribosomal small subunit assembly, while downregulated genes were enriched in respiratory electron transport chain, mitochondrial respiratory chain complex assembly, and aerobic respiration (S3B Fig). For AKI-CKD + EGT vs AKI-CKD comparisons, the upregulated genes showed enrichment in mitochondrial electron transport chain and mitochondrial respiratory chain complex assembly, whereas downregulated genes were involved in translation, ribosomal small subunit assembly, and mitochondrial respiratory chain complex assembly (S3B Fig). Furthermore, compared with the control group, 17 genes were downregulated in the AKI-CKD group and subsequently upregulated after EGT treatment, while 30 genes showed the opposite trend (upregulated in AKI-CKD and downregulated post-EGT) (Fig 4C). The expression patterns of these genes across the three groups are presented as a heatmap in Fig 4D. Functional enrichment analysis revealed their involvement in cytoplasmic translation, proton motive force-driven mitochondrial ATP synthesis, translation (both presynaptic and postsynaptic), and electron transport-coupled proton transfer (Fig 4E). These findings suggest that AKI-to-CKD progression is associated with dysregulation of mitochondria-related transcriptional programs in LOH cells, whereas EGT treatment is associated with partial normalization of these transcriptional alterations. Additionally, we observed compromised translational function in LOH cells, evidenced by significant differential expression of ribosomal-related genes (Figs 4C-D). To identify potential transcriptional regulators of these translation- and mitochondria-associated genes, we analyzed differentially expressed TFs in LOH cells. Notably, Batf3, Bcl11a, Irf8, and Irf5 exhibited an initial upregulation followed by downregulation across the Control, AKI-CKD, and AKI-CKD + EGT groups, whereas Mafg, Bclaf1, and Nfia displayed an opposite trend (Fig 4F). Subsequent analysis of their target genes revealed a regulatory network (Fig 4G), with Cst3, Cyp4b1, and Malat1 being closely associated with oxidative phosphorylation (Fig 4H). In summary, our results suggest that LOH cells in AKI-to-CKD mice exhibit mitochondria-related transcriptional dysregulation, and that EGT treatment may contribute to partial preservation of mitochondrial homeostasis in this cell population.

### 3.5. EGT treatment is associated with partial preservation of mitochondria-related transcriptional programs in PT cells during AKI-to-CKD progression

**Fig 5:**
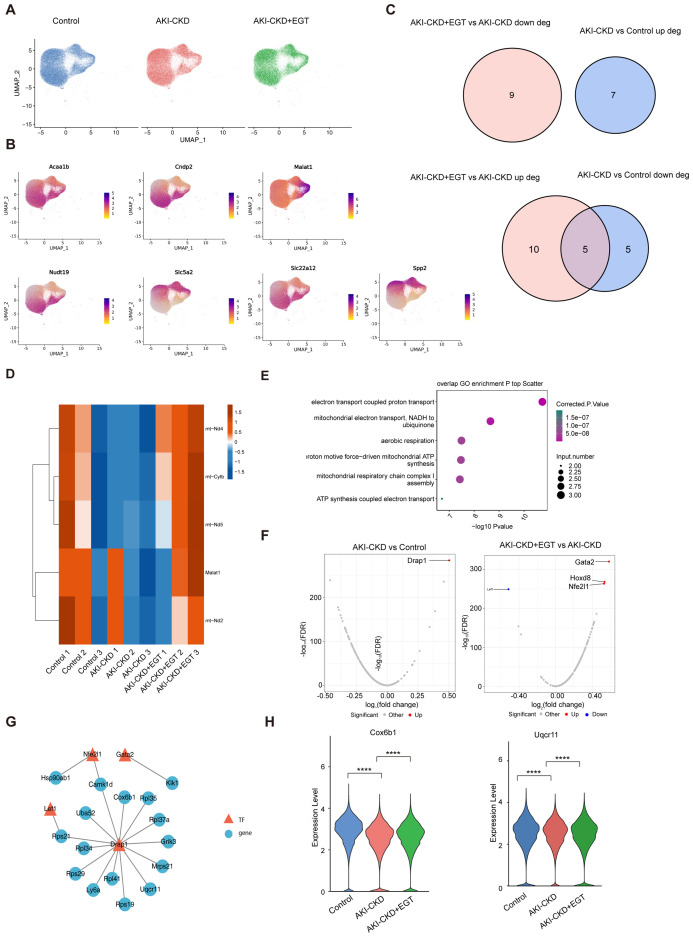
EGT treatment is associated with partial preservation of mitochondria-related transcriptional programs in PT cells during AKI-to-CKD progression. **(A)** UMAP plot of PT cells. Colors indicate sample groups. **(B)** UMAP plot showing the expression levels of marker genes in PT cells. **(C)** The Venn diagram shows overlapping results of down-regulated genes in AKI-CKD + EGT vs AKI-CKD and up-regulated genes in AKI-CKD vs Control, up-regulated genes in AKI-CKD + EGT vs AKI-CKD and down-regulated genes in AKI-CKD vs Control. **(D)** The heatmap diagram showing the expression profile of overlapped DEGs in **C. (E)** The bubble plot showing the most enriched GO results of overlapped DEGs in **C. (F)** Volcano plot showing differential transcription regulons in two comparison groups (AKI-CKD vs Control and AKI-CKD + EGT vs AKI-CKD). Blue represents down-regulation, red represents up-regulation. **(G)** Network showing four DE-TFs and their target DEGs. **(H)** Violin plot showing the expression levels of Cox6b1 and Uqcr11 in each sample group in PT cells.

To investigate the mechanism by which EGT affects PT cells during AKI-to-CKD progression, we isolated PT cells for further analysis. UMAP analysis revealed spatial distribution differences in PT cells between the AKI-CKD and Control groups, with partial restoration toward the normal state following EGT treatment (Fig 5A). The UMAP distribution of PT cell marker genes, Acaa1b, Cndp2, Malat1, Nudt19, Slc5a2, Slc22a12, and Spp2, is shown in Fig 5B. Differential gene expression analysis of PT cells across the three groups was performed. Volcano plots illustrating DEGs in AKI-CKD vs. Control and AKI-CKD + EGT vs. AKI-CKD are presented in S4A Fig. Functional enrichment analysis demonstrated that in AKI-CKD vs Control comparisons, upregulated genes were primarily associated with translation at presynapse, translation at postsynapse, and cytoplasmic translation, while downregulated genes were enriched in regulation of protein localization, positive regulation of protein import into nucleus, cellular response to heat, positive regulation of cell size, ATP synthesis coupled electron transport, mitochondrial respiratory chain complex I assembly, proton motive force−driven mitochondrial ATP synthesis, aerobic respiration, mitochondrial electron transport, NADH to ubiquinone, and electron transport coupled proton transport (S4B Fig). For AKI-CKD + EGT vs AKI-CKD comparisons, the upregulated genes showed enrichment in electron transport coupled proton transport, aerobic respiration, proton motive force−driven mitochondrial ATP synthesis, mitochondrial electron transport, NADH to ubiquinone, ATP synthesis coupled electron transport, mitochondrial respiratory chain complex I assembly, mitochondrial electron transport, cytochrome c to oxygen, respiratory electron transport chain, positive regulation of apoptotic process, and negative regulation of apoptotic process, whereas downregulated genes were involved in rRNA processing, ribosomal small subunit biogenesis, translation at postsynapse, translation at presynapse, ribosomal small subunit assembly, maturation of SSU − rRNA, translation, and cytoplasmic translation (S4B Fig). Furthermore, compared with the control group, 5 genes were downregulated in the AKI-CKD group and subsequently upregulated after EGT treatment (Fig 5C). The expression patterns of these genes across the three groups are presented as a heatmap in Fig 5D. Functional enrichment analysis revealed their involvement in electron transport coupled proton transport, mitochondrial electron transport NADH to ubiquinone, aerobic respiration, proton motive force-driven mitochondrial ATP synthesis, mitochondrial respiratory chain complex I assembly, and ATP synthesis coupled electron transport (Fig 5E). These findings suggest that AKI-to-CKD progression is associated with dysregulation of oxidative phosphorylation- and electron transport chain-related transcriptional programs in PT cells, whereas EGT treatment is associated with partial normalization of these mitochondria-related transcriptional alterations. To identify potential transcriptional regulators of these translation- and mitochondria-associated genes, we analyzed differentially expressed TFs in PT cells (Fig 5F). Subsequent analysis of their target genes revealed a regulatory network (Fig 5G), with Cox6b1 and Uqcr11 being closely associated with oxidative phosphorylation, and their specific expression trends across the three groups are shown in Fig 5H. Cytochrome c oxidase subunit 6B1 (Cox6b1) is one of the subunits of the cytochrome c oxidase (COX). The principal role of Cox6b1 is to connect two COX monomers to form a dimer, serving a role in the cellular respiratory chain [30]. Uqcr11, a key subunit of the mitochondrial respiratory chain complex, is integral to maintaining mitochondrial function and integrity. It contributes to the regulation of the mitochondrial network, energy metabolism, and cellular oxidative phosphorylation processes [31]. In summary, our results suggest that PT cells in AKI-to-CKD mice exhibit mitochondria-related transcriptional dysregulation, and that EGT treatment may help preserve mitochondrial homeostasis in this tubular compartment.

### 3.6. EGT alleviates mitochondrial injury-related phenotypes in renal tubular epithelial cells

**Fig 6:**
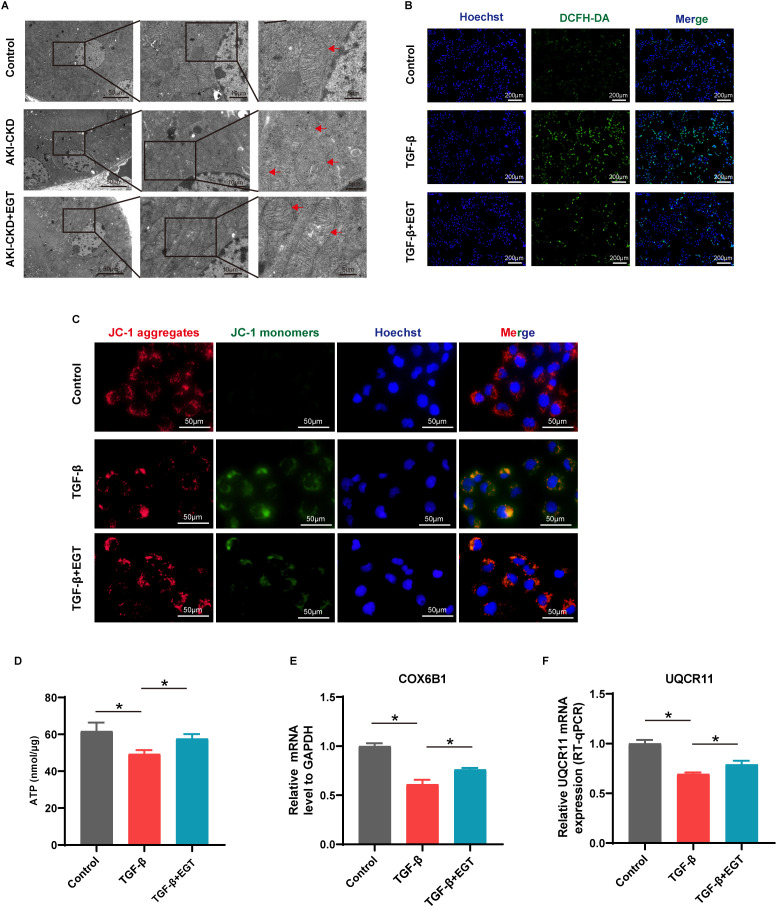
EGT alleviates mitochondrial injury-related phenotypes in renal tubular epithelial cells. **(A)** Representative transmission electron microscopy images of renal tissues from different groups, showing mitochondrial ultrastructure. Scale bar = 50 μm, 10 μm, 5 μm. **(B)** Intracellular ROS levels detected by DCFH-DA fluorescence. Scale bar = 200 μm. **(C)** Mitochondrial membrane potential measured by JC-1 staining. Scale bar = 50 μm. **(D)** ATP content determined by bioluminescence assay. **(E–F)** Relative mRNA expression levels of UQCR11 and COX6B1 in HK-2 cells. Data are expressed as mean ± SD, n = 3, *P < 0.05; **P < 0.01; ***P < 0.001.

**Fig 7:**
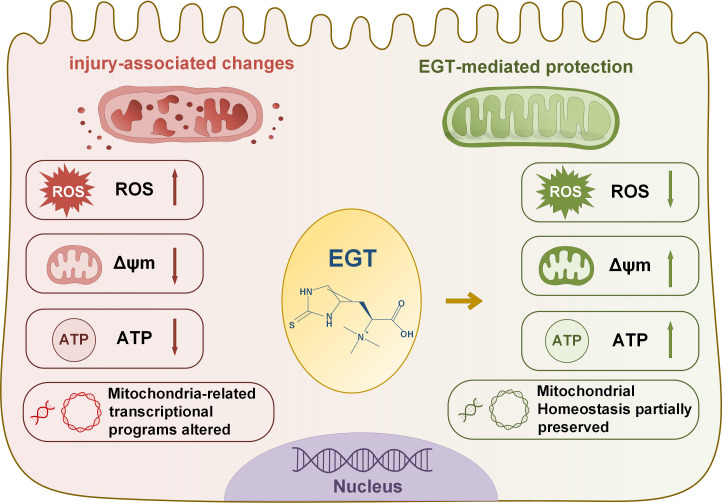
Proposed mechanism by which ergothioneine improves mitochondrial function and attenuates renal fibrosis.

To further validate the impact of EGT on mitochondria, we performed transmission electron microscopy on renal tissues. The results revealed severe ultrastructural damage to mitochondria in cells of the AKI-CKD group kidneys, including fragmented and dissolved cristae and marked vacuolization. In contrast, EGT treatment attenuated these ultrastructural abnormalities, as reflected by better-preserved mitochondrial morphology and reduced vacuolization (Fig 6A). Consistently, in vitro experiments using injured HK-2 cells showed that EGT treatment reduced ROS overproduction, partially preserved mitochondrial membrane potential, and increased cellular ATP levels (Fig 6B-D). These findings collectively suggest that EGT alleviates mitochondrial injury-related phenotypes in renal tubular epithelial cells under injury conditions.UQCR11 and COX6B1, the human homologues of the mitochondria-related genes highlighted by the scRNA-seq analysis, were further validated in HK-2 cells by qPCR. Consistent with the scRNA-seq findings, the mRNA levels of UQCR11 and COX6B1 were decreased in injured HK-2 cells, whereas EGT treatment partially restored their expression (Fig 6E-F). These validation results support the reliability of the transcriptomic findings and suggest that EGT treatment is associated with partial preservation of mitochondria-related molecular features involved in oxidative phosphorylation and electron transport chain regulation. A schematic summary of the proposed mechanism underlying EGT-associated mitochondrial protection during AKI-to-CKD progression is presented in Fig 7.

Ergothioneine may alleviate injury-associated mitochondrial dysfunction by reducing ROS accumulation, restoring mitochondrial membrane potential and ATP production, and partially preserving mitochondria-related transcriptional programs, thereby contributing to the attenuation of renal fibrotic progression.

## 4. Discussion

In this study, we found that EGT attenuated renal injury and fibrosis in a cisplatin-induced AKI-to-CKD model and was associated with improved mitochondria-related features in renal tubular epithelial cells. Through integrated histopathological, biochemical, single-cell transcriptomic, and in vitro analyses, our findings suggest that EGT exerts renoprotective effects that are associated with improved mitochondria-related transcriptional and injury-related phenotypic features, thereby contributing to the attenuation of renal fibrosis and functional decline.

During AKI-to-CKD progression, mitochondrial dysfunction has been identified as a central pathological mechanism [32]. Renal tubular epithelial cells are rich in mitochondria and rely on oxidative phosphorylation to generate substantial ATP to support their high-energy-demand functions such as reabsorption and secretion [33]. Under AKI conditions, insults such as ischemia or toxins cause structural damage and functional disruption of mitochondria, manifesting as reduced ATP synthesis, excessive ROS production, impaired assembly of electron transport chain complexes, and diminished oxidative phosphorylation capacity [34]. These alterations not only directly trigger apoptosis and necrosis but also promote inflammatory responses, fibrotic processes, and impaired cellular repair through metabolic reprogramming, such as suppression of fatty acid β-oxidation and enhanced glycolysis, thereby driving maladaptive AKI-to-CKD progression [35]. Our single-cell transcriptomic analysis further suggests that AKI-to-CKD is associated with dysregulation of mitochondria-related transcriptional programs, particularly those involving electron transport, ATP synthesis, and respiratory chain assembly, in DCT, LOH, and PT cells. EGT intervention was associated with partial normalization of these mitochondria-related transcriptional alterations and with attenuation of renal functional decline and fibrosis progression.

Notably, we identified a conserved set of mitochondria-related genes—including Cst3, Cyp4b1, Malat1, Cox6b1, and Uqcr11—that were consistently dysregulated across different tubular segments during AKI-to-CKD progression and were partially normalized following EGT treatment. Cst3 is a 13-kDa non-glycosylated protein comprising 120 amino acids. As a member of the Cystatin family, it functions as an endogenous inhibitor of cysteine proteases and was initially recognized for its role in kidney diseases [36]. Studies have demonstrated that Cst3 exerts broad inhibitory effects on oxidative stress-induced cell death. Oxidative stress triggers the leakage of lysosomal cysteine proteases into the cytosol, leading to cell death. However, oxidative stress also induces the synthesis of Cst3 mRNA and the production of Cst3 protein. If the amount of Cst3 produced is sufficient to inhibit protease activity, it can prevent cell death [37]. Cyp4b1 is an enigmatic mammalian cytochrome P450 (Cyp450) monooxygenase. Within the Cyp450 superfamily, several enzymes are closely associated with oxidative stress and mitochondrial function. Cyp2e1 is a key inducer of intracellular oxidative stress, significantly increasing the production of ROS and reactive nitrogen species (RNS). This leads to mitochondrial DNA damage, post-translational protein modifications, and lipid peroxidation, ultimately causing mitochondrial dysfunction [38]. Cyp1b1, upon mitochondrial targeting, mediates the metabolic activation of polycyclic aromatic hydrocarbons (PAHs), leading to excessive mitochondrial reactive oxygen species (ROS) generation, resulting in mtDNA damage, respiratory chain dysfunction, and oxidative stress [39]. The physiological function of CYP4B1 in humans remains unclear, and it is therefore classified as an orphan P450. The relationship between Cyp4b1 and oxidative stress has not yet been investigated and represents a key direction for future research [40]. Long non-coding RNAs (lncRNAs) are RNA molecules that do not possess protein-coding potential. Nevertheless, this RNA subgroup has been demonstrated to play crucial roles in the regulation of oxidative stress and other biological processes. LncRNA Malat1 acts as a molecular sponge for miR-26b-5p to upregulate Mfn1 expression, thereby suppressing mitochondrial fission and reactive oxygen species production, maintaining mitochondrial dynamics homeostasis, and ultimately reducing oxidative stress and mitochondrial-dependent apoptosis [41]. Furthermore, LncRNA Malat1 interacts with the RNA-binding protein FUS to stabilize Acsf2 mRNA, leading to mitochondrial iron accumulation and lipid peroxidation, thereby triggering ferroptosis and exacerbating oxidative stress and mitochondrial dysfunction in septic acute kidney injury [42]. Cytochrome oxidase subunit 6B1 (Cox6b1) is a subunit of cytochrome oxidase. Altered Cox6b1 expression may affect cytochrome c oxidase function and may contribute to disease development. Cox6b1 alleviates hypoxia/reoxygenation-induced oxidative stress injury in cardiomyocytes by maintaining mitochondrial function, reducing ROS production, and inhibiting mitochondrial permeability transition pore (mPTP) opening [43]. Uqcr11 is a core subunit of mitochondrial complex III, which is responsible for transferring electrons from coenzyme Q to cytochrome c in the mitochondrial electron transport chain and is essential for maintaining normal oxidative phosphorylation. Uqcr11 effectively reduces the level of ROS in mitochondria by enhancing the activity of antioxidant enzymes such as SOD2 and CAT, thereby alleviating oxidative stress. Furthermore, Uqcr11 helps maintain mitochondrial membrane potential and functional integrity, reducing the release of pro-apoptotic factors. Its expression level is positively correlated with cellular antioxidant capacity, making it a key molecule in regulating intracellular redox balance [31]. In the present study, qPCR analyses further validated the EGT-associated partial restoration of UQCR11 and COX6B1 expression in HK-2 cells, supporting the reliability of the transcriptomic findings and suggesting their potential involvement in EGT-associated mitochondrial protection during tubular injury.

However, several limitations of our study should be acknowledged. First, although the cisplatin-induced AKI-to-CKD model is well-established and widely used, it may not fully recapitulate the heterogeneity of human AKI etiologies, which include ischemia-reperfusion injury, sepsis, and nephrotoxin exposure. Future studies should validate EGT’s efficacy in additional AKI models to ensure broad applicability. Second, although scRNA-seq provides high-resolution cellular profiling, functional validation of the identified genes and pathways, for example through genetic knockout, knockdown, or overexpression experiments, would strengthen our mechanistic conclusions. Third, mitochondrial respiration was not directly assessed using oxygen consumption-based assays, such as Seahorse analysis. Although TEM, ROS measurement, JC-1 staining, ATP quantification, and scRNA-seq analysis collectively supported an improvement in mitochondria-related status after EGT treatment, these approaches do not directly quantify mitochondrial respiratory capacity. Therefore, conclusions regarding mitochondrial functional recovery should be interpreted with appropriate caution. Future studies incorporating direct bioenergetic measurements will be required to further define the effects of EGT on mitochondrial respiration. Finally, although our study focused on tubular cells, other cell types such as fibroblasts and endothelial cells may also contribute to EGT’s renoprotective effects and warrant examination in future research.

In conclusion, our results support EGT as a potential therapeutic candidate for mitigating AKI-to-CKD progression, in association with improved mitochondria-related status in renal tubular cells. These findings not only deepen our understanding of the pathological mechanisms underlying CKD progression but also highlight the potential of mitochondrial-focused therapies in preventing chronic kidney disease.

## 5. Conclusions

This study suggests that ergothioneine (EGT) may attenuate AKI-to-CKD progression and is associated with improved mitochondria-related status in renal tubular epithelial cells. Single-cell transcriptomic analysis identified EGT-associated changes in mitochondria-related pathways across DCT, LOH, and PT cell populations, while TEM, ROS measurement, JC-1 staining, and ATP assays further supported a protective effect of EGT on mitochondrial injury-related phenotypes. Collectively, these findings support EGT as a potential mitochondria-related therapeutic candidate for slowing maladaptive renal repair and CKD progression.

## Supporting information

S1 Fig Graphical abstract. (TIF)

S2 Fig EGT treatment is associated with partial preservation of mitochondria-related transcriptional programs in DCT cells during AKI-to-CKD progression. (A)Volcano plots showing differentially expressed genes (DEGs) in DCT cells for the AKI-CKD vs Control and AKI-CKD + EGT vs AKI-CKD comparisons. (B)Bar plots showing the top 10 enriched GO biological process terms for upregulated and downregulated genes in the AKI-CKD vs Control and AKI-CKD + EGT vs AKI-CKD comparisons in DCT cells.
(TIF)

S3 Fig EGT treatment is associated with partial preservation of mitochondria-related transcriptional programs in LOH cells during AKI-to-CKD progression. (A) Volcano plots showing differentially expressed genes (DEGs) in LOH cells for the AKI-CKD vs Control and AKI-CKD + EGT vs AKI-CKD comparisons. (B) Bar plots showing the top 10 enriched GO biological process terms for upregulated and downregulated genes in the AKI-CKD vs Control and AKI-CKD + EGT vs AKI-CKD comparisons in LOH cells.
(TIF)

S4 Fig EGT treatment is associated with partial preservation of mitochondria-related transcriptional programs in PT cells during AKI-to-CKD progression. (A) Volcano plots showing differentially expressed genes (DEGs) in PT cells for the AKI-CKD vs Control and AKI-CKD + EGT vs AKI-CKD comparisons. (B) Bar plots showing the top 10 enriched GO biological process terms for upregulated and downregulated genes in the AKI-CKD vs Control and AKI-CKD + EGT vs AKI-CKD comparisons in PT cells.
(TIF)

## Abbreviations

PT: Proximal tubule
DT: Distal tubule
LOH: Loop of Henle
TF: Transcription factor
AKI: Acute kidney injury
CKD: Chronic kidney disease
COX: cytochrome c oxidase
DEG: differentially expressed genes
GFR: glomerular filtration rate
Cox6b1: Cytochrome c oxidase subunit 6B1
Cyp450: cytochrome P450
ROS: reactive oxygen species
RNS: reactive nitrogen species
TFs: transcription factors
mPTP: mitochondrial permeability transition pore
