# Supplementary figures and images for: Single-cell transcriptomics identifies ergothioneine as a mitochondrial protector to prevent AKI-to-CKD progression
Source: PLoS One. 2026 Jun 23;21(6):e0351630. doi: 10.1371/journal.pone.0351630 (PMC13289929; doi:10.1371/journal.pone.0351630)

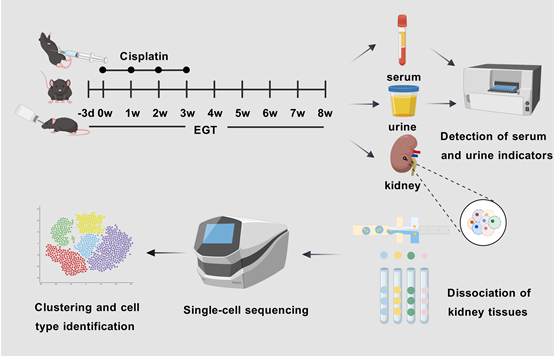

Supplement: S1 Fig — (TIF) [file pone.0351630.s001.tif]

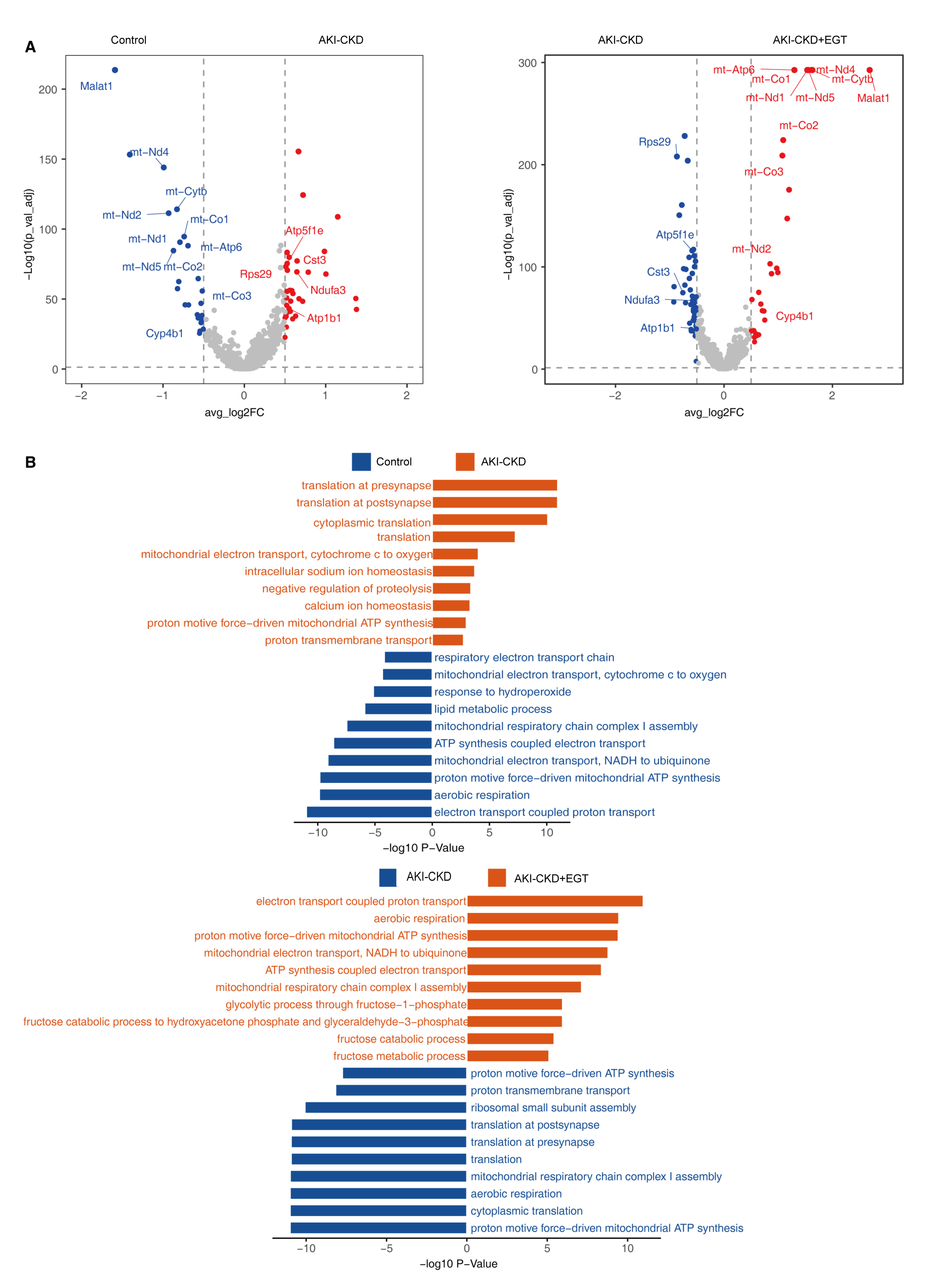

Supplement: S2 Fig — (A)Volcano plots showing differentially expressed genes (DEGs) in DCT cells for the AKI-CKD vs Control and AKI-CKD + EGT vs AKI-CKD comparisons. (B)Bar plots showing the top 10 enriched GO biological process terms for upregulated and downregulated genes in the AKI-CKD vs Control and AKI-CKD + EGT vs AKI-CKD comparisons in DCT cells. (TIF) [file pone.0351630.s002.tif]

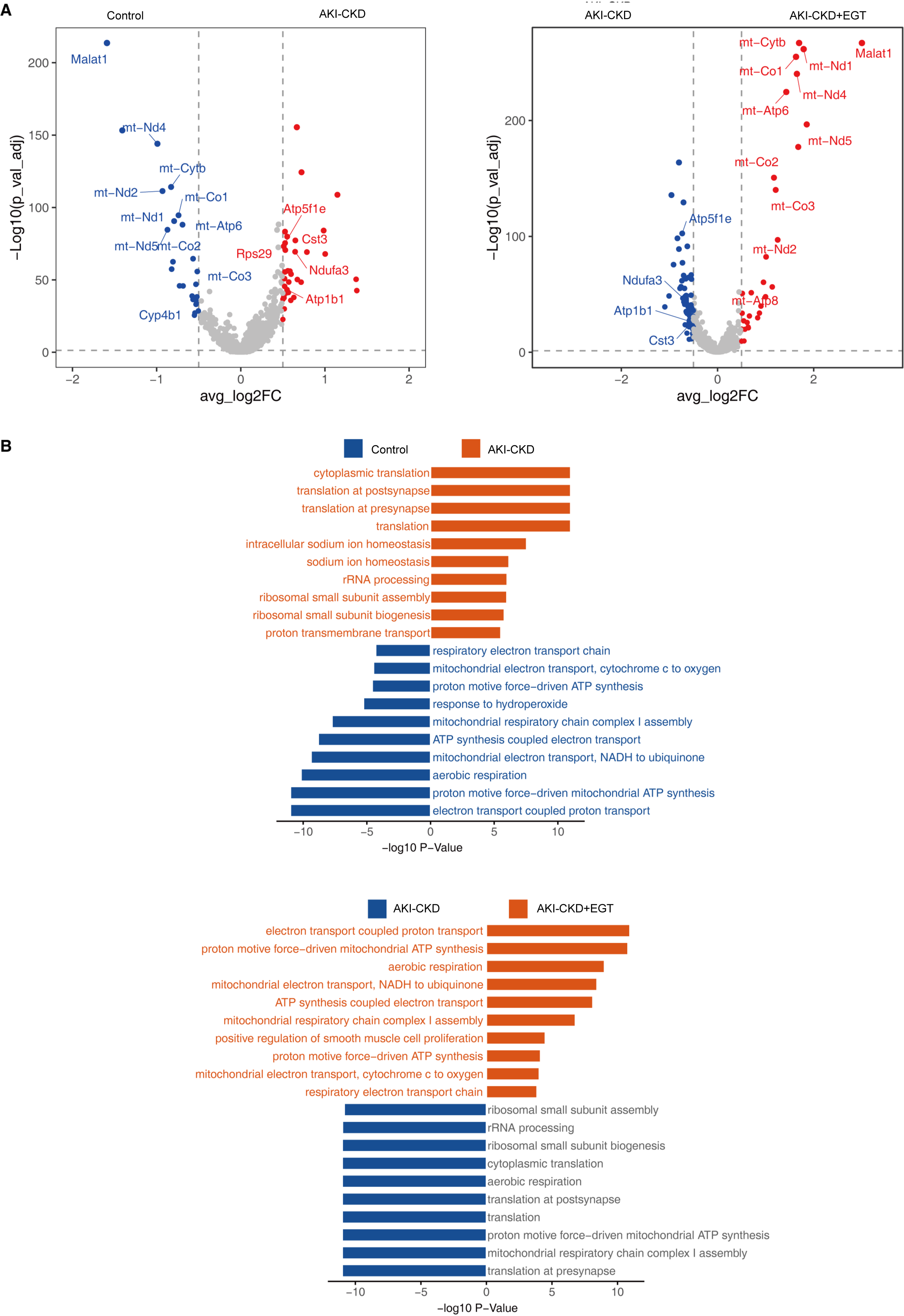

Supplement: S3 Fig — (A) Volcano plots showing differentially expressed genes (DEGs) in LOH cells for the AKI-CKD vs Control and AKI-CKD + EGT vs AKI-CKD comparisons. (B) Bar plots showing the top 10 enriched GO biological process terms for upregulated and downregulated genes in the AKI-CKD vs Control and AKI-CKD + EGT vs AKI-CKD comparisons in LOH cells. (TIF) [file pone.0351630.s003.tif]

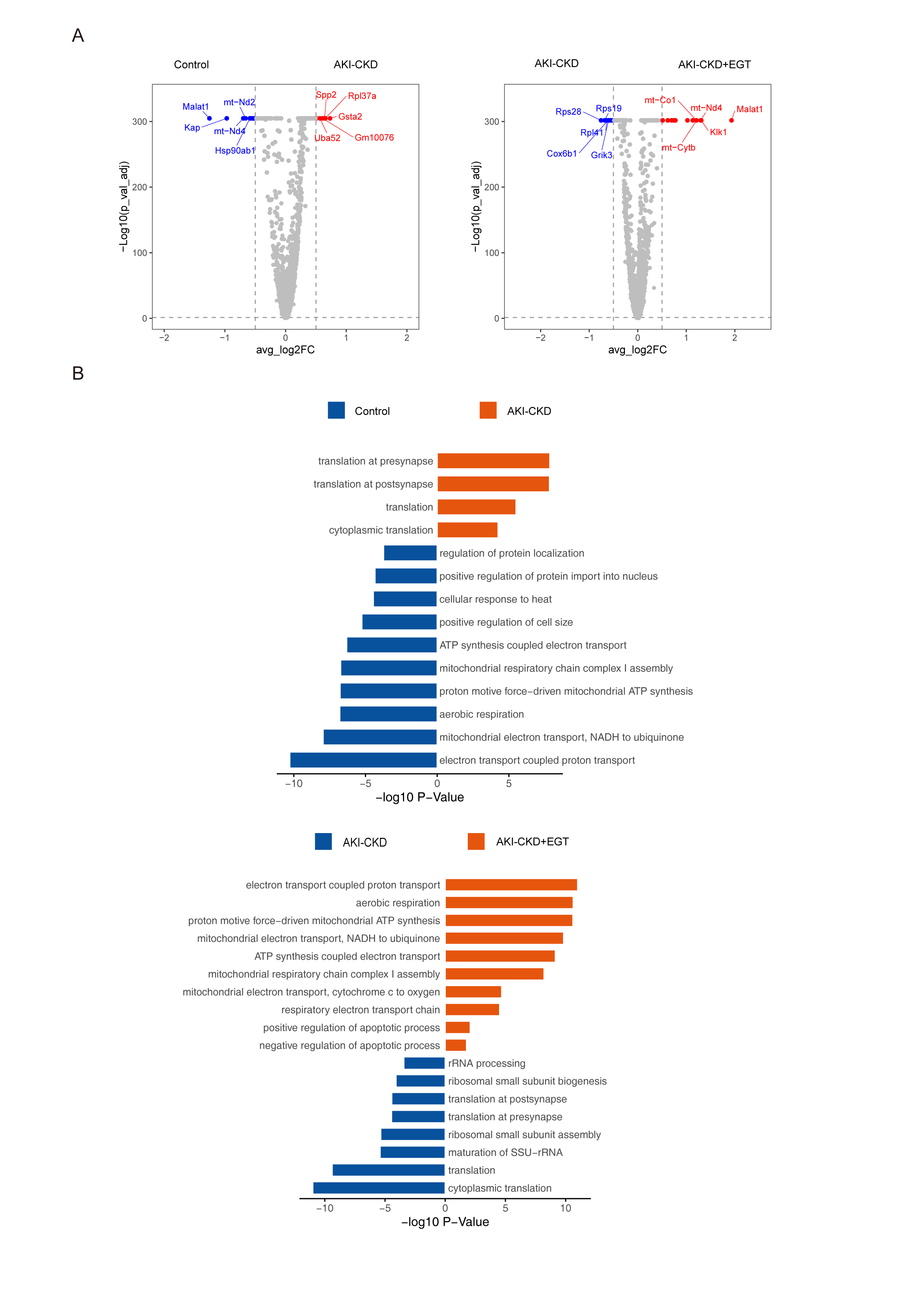

Supplement: S4 Fig — (A) Volcano plots showing differentially expressed genes (DEGs) in PT cells for the AKI-CKD vs Control and AKI-CKD + EGT vs AKI-CKD comparisons. (B) Bar plots showing the top 10 enriched GO biological process terms for upregulated and downregulated genes in the AKI-CKD vs Control and AKI-CKD + EGT vs AKI-CKD comparisons in PT cells. (TIF) [file pone.0351630.s004.tif]
